# Capability versus health: a comparison of the ICECAP-O and EQ-5D-5L in elderly Chinese patients with Kashin-Beck disease

**DOI:** 10.1186/s12955-026-02595-9

**Published:** 2026-08-07

**Authors:** Qian Fang, Jiayou Fu, Hongyan Li, Jiawei Li, Bo Li, Jie Liu

**Affiliations:** 1 Huaihe Hospital of Henan University, Kaifeng, 475000 China; 2 School of Nursing and Health, Henan University, Kaifeng, 475000 China; 3 Xi’an Lintong District Center for Disease Control and Prevention, Xi’an, 710600 China

**Keywords:** ICECAP-O, EQ-5D-5L, Kashin-Beck disease, Elderly, China, Quality of life

## Abstract

**Objectives:**

This study aimed to compare the performance of the Chinese versions of the ICECAP-O and EQ-5D-5L in patients with Kashin-Beck disease (KBD), and to explore the relationship between the two instruments.

**Methods:**

Data were collected from 222 elderly Chinese patients with KBD using both the ICECAP-O and the EQ-5D-5L. To compare the two instruments and examine their relationship, we assessed floor/ceiling effects, known-groups validity (non-parametric tests with a priori hypotheses), polychoric correlations, and regression analyses.

**Results:**

The Chinese version of the ICECAP-O was free of floor or ceiling effects and demonstrated known-groups validity consistent with a priori hypotheses. In contrast, the EQ-5D-5L exhibited ceiling effects in the Mobility, Self-care, Usual activities, and Anxiety/Depression dimensions, and no floor effects. The median (Q1, Q3) index scores were 0.6465 (0.5558, 0.7609) for the ICECAP-O and 0.6050 (0.3755, 0.7690) for the EQ-5D-5L. A moderate-to-strong positive polychoric correlation was found between the two instrument scores (*ρ* = 0.656, *p* < 0.001). Regression analysis showed that both instrument scores differed across subgroups defined by sociodemographic characteristics and clinical conditions.

**Conclusions:**

This study provides the first application of the ICECAP-O in patients with KBD in China. The instrument captures capability well-being dimensions beyond the EQ-5D-5L. These findings support the complementary use of both instruments for a more comprehensive assessment of patient well-being and to inform economic evaluations.

## Introduction

Kashin-Beck disease (KBD) is a chronic and disabling osteoarthropathy that primarily affects the bones and cartilage of major joints [1, 2]. KBD was first discovered and reported in 1849 by I. M. Yurenski, a Russian land surveyor, in the Urov River valley (Transbaikal region, Russian Far East). It has remained endemic for over 150 years across a wide geographic belt, stretching from Siberia in Russia through Northeast China to Southwest China [3]. The disease pathologically targets the articular cartilage and growth plates, leading to joint pain, deformity, and functional impairment [4]. Due to its progressive nature and tendency to cause severe disability, KBD imposes a higher overall burden on patients than most other bone and joint diseases [5].

Historically, KBD was endemic across 377 counties in 13 provinces of China [6]. Although preventive strategies such as selenium supplementation and improved water sources have significantly reduced its incidence [7], the disease remains persistent. Notably, Shaanxi Province bears the highest caseload in the country. According to the *China Health Statistics Yearbook 2022* [8], it had an estimated 56,970 patients with Kashin-Beck disease as of 2021, accounting for nearly 30% of China’s total. Within the province, several endemic foci exist, among which a key area is located within the provincial capital, Xi’an. Here, cases are predominantly clustered in the mountainous Lishan region and the adjacent loess tableland. Therefore, this area was selected as the study site.

Previous studies assessing the quality of life in KBD patients have predominantly employed instruments such as the EQ-5D [9], SF-36 [10], WOMAC [11], and the Kashin-Beck Disease Quality of Life (KBDQOL) [12], which focus on physical symptoms and functional capacity. This disease-centric perspective, however, frequently overlooks the broader psychosocial dimensions of the illness experience, including emotional well-being, social participation, and the sense of meaning in life. Supporting this concern, a recent study reported that 53.2% of KBD patients exhibited depressive symptoms—a prevalence markedly higher than that in patients with generalized osteoarthritis [13].

To address this gap, this study is grounded in the Capability Approach, a framework pioneered by Nobel laureate Amartya Sen [14], which conceptualizes development as the expansion of individuals’ substantive freedoms and opportunities. Building on this framework, several capability-based measures have been developed, including the ICECAP-A [15] and the ICECAP-O [16]. The ICECAP-O has been extensively validated in several countries, including the United Kingdom [16, 17], Canada [18, 21, 23], and the Netherlands [19]. It demonstrates good reliability and validity, with the ability to distinguish between groups with different health conditions (e.g., hip fracture [20, 21]) and mental well-being (e.g., depression [22]).

Since new cases of KBD have become rare, the existing patients are predominantly elderly [23]. These patients have long disease durations and severe joint deformities, and most face limitations in mobility and self-care. In addition, many of them live in mountainous areas, which further restricts their social participation and reduces social support. Consequently, on top of physical limitations, they also experience constraints on their substantive freedom to choose among different ways of living — which is precisely the core concept of capability. Therefore, assessing capability is as important as measuring physical health in these elderly patients. However, the measurement performance of the health-focused EQ-5D-5 L and the capability-focused ICECAP-O in KBD patients, as well as the relationship between the two instruments, has not yet been investigated. Hence, this study aims to compare the measurement performance of the two tools and explore their association, so as to provide a more comprehensive basis for the assessment of this population.

## A priori hypotheses

Based on the capability approach [14] and previous empirical studies [18–20, 30], we formulated the following a priori hypotheses to guide interpretation of the results:

(1) The total scores of ICECAP-O and EQ-5D-5L are expected to show a moderate positive correlation (0.4 < *ρ* < 0.7), as health status influences but does not fully determine capability well-being.

(2) Across dimension pairs, the strongest negative correlation is expected between ICECAP-O Control and EQ-5D-5L Usual activities/Mobility, due to opposite scoring directions and because physical functioning underpins personal autonomy. The weakest correlation is expected between ICECAP-O Attachment and EQ-5D-5L Self-care, reflecting the conceptual distinctness of emotional belonging from basic physical function.

(3) For known-groups validity, we hypothesize that higher education (compared with illiteracy), being married (compared with unmarried/divorced/widowed), not living alone (compared with living alone), and less severe disease (Grade I vs. Grade II/III) are each associated with higher scores on both instruments, based on prior KBD literature [5, 9, 13] and general geriatric studies.

Both the ICECAP-O and EQ-5D-5L are formative measures, where items define rather than reflect the underlying construct. Therefore, traditional psychometric assessments based on reflective measurement theory (e.g., internal consistency, factor analysis) are not applicable. Instead, we evaluate the instruments through polychoric correlation patterns, group comparisons, and regression analyses.

## Methods

### Study design and participants

This cross-sectional study, conducted between January and May 2025, recruited participants aged ≥ 65 years with a registry-confirmed diagnosis of KBD from four subdistricts (Xiekou, Renzong, Xiaojin, and Muzhai) in Lintong District, Xi’an, China. A cluster sampling method was used to recruit participants from these subdistricts.

The inclusion criteria were: (1) diagnosis of stage I or higher KBD according to the Chinese National Standard “Diagnosis of KBD ” (WS/T 207‑2010) [24]; (2) ability to communicate effectively; and (3) provision of written informed consent.

The exclusion criteria included: (1) presence of other chronic osteoarticular diseases that could confound pain and function assessment, such as primary osteoarthritis or spinal arthritis; and (2) severe cognitive impairment or psychiatric disorders that would prevent reliable communication or completion of the questionnaires.

Ethical approval for this study was granted by the Ethics Committee of the School of Nursing and Health, Henan University. Written informed consent was obtained from all participants prior to their involvement in the study. Further details are provided in the Ethics Statement section.

### Data collection and quality control

Data collection was conducted via face-to-face interviews administered by a team of interviewers who received standardized training. The training lasted two days and included: understanding the meaning and response levels of each item in the ICECAP-O and EQ-5D-5L; practicing the use of the Shaanxi dialect to read each question and response option clearly and item by item; and simulating real interview scenarios to learn how to handle participants who might hesitate or give off-topic answers.

After training, the interviewers conducted face-to-face structured questionnaire surveys in the participants’ communities with the assistance of local community health service centers. The specific procedure was as follows: the interviewers read each question and its response options aloud to the elderly patients one by one using the Shaanxi dialect; the participants answered verbally; and the interviewers recorded the answers on paper questionnaires. Data were collected on sociodemographic characteristics (including sex, age, body mass index [BMI], education level, marital status, and living alone status), clinical conditions of KBD (including disease severity and duration), along with the ICECAP-O and EQ-5D-5L instruments.

### Instruments

The KBD patients in this study area were predominantly elderly; therefore, the ICECAP-O instrument, which was specifically designed for individuals aged 65 years and older, was selected. Building on Grewal [25] et al.’s work, Coast et al. (2008) developed this measure, which assesses capability well-being. Its Chinese version has been validated for use in the elderly Chinese population by Xiong et al. [26].

In this study, capability well-being was assessed using the ICECAP-O instrument. It evaluates five distinct dimensions: Attachment (love and friendship), Security (thinking about the future without concern), Role (doing things that make you feel valued), Enjoyment (enjoyment and pleasure), and Control (independence). Each dimension is rated on a four-level scale from 1 (no capability) to 4 (full capability). Subsequently, the responses were converted into a tariff score (range: 0 ~ 1) by applying the dimension-weighting coefficients derived by Flynn et al. [27] through best-worst scaling. A higher tariff score indicates a greater level of capability well-being, reflecting greater freedom to live a valued life.

The EQ-5D-5 L (EuroQol Group [28]) is a widely used, generic instrument for assessing health-related quality of life. It consists of two parts: a descriptive system for health states and a visual analogue scale (EQ-VAS). The descriptive system assesses five dimensions: Mobility, Self-Care, Usual Activities, Pain/Discomfort, and Anxiety/Depression. Each dimension has five levels of severity: no problems, slight problems, moderate problems, severe problems, and extreme problems (scored from 1 to 5, respectively). The descriptive system yields 3,125 possible health states. For this study, utility scores for these states were calculated by applying the Chinese value set developed by Luo et al. [29], resulting in utility scores ranging from − 0.391 (worse than death) to 1.000 (full health).

Due to the advanced age of the cohort, the EQ-VAS was employed solely for supplementary descriptive analysis. The utility scores derived from the descriptive system served as the primary, objective measure for all inferential analyses.

### Statistical analysis

Data were analyzed using SPSS 25.0 and Mplus 8.0. Descriptive statistics were used to summarize the sociodemographic characteristics and clinical conditions of the KBD patients, as well as their scores (ICECAP-O and EQ-5D-5L). Categorical variables are presented as *n* (%). Continuous data are summarized as mean ± standard deviation (SD) if normally distributed, or as median (first quartile [Q1], third quartile [Q3]) otherwise.

Floor and ceiling effects were examined by calculating the proportion of responses at the highest and lowest levels for each dimension of both instruments.

Polychoric correlation coefficients (*ρ*) were calculated between the total scores and across all dimension pairs of the two instruments. Statistical significance was set at *p* < 0.05. Known-groups validity was examined by testing Hypothesis 3, which states that higher education, being married, not living alone, and less severe disease were expected to be associated with higher scores. Differences across subgroups were examined using the Mann-Whitney *U* test or Kruskal-Wallis *H* test as appropriate. Effect sizes for group comparisons were calculated as rank-biserial correlation (*r*) for Mann-Whitney *U* tests and as eta-squared (*η²*) for Kruskal-Wallis *H* tests, and were interpreted according to Cohen’s conventions (small: *r* ≥ 0.10, *η²* ≥ 0.01; medium: *r* ≥ 0.30, *η²* ≥ 0.06; large: *r* ≥ 0.50, *η²* ≥ 0.14).

Multiple ordinary least squares regression with robust standard errors was applied to identify sociodemographic characteristics and clinical conditions predictors of ICECAP‑O tariff scores and EQ‑5D‑5L utility scores. Multicollinearity was assessed using the variance inflation factor (VIF), with values exceeding 10 considered indicative of potential multicollinearity. Model assumptions, such as linearity and homoscedasticity, were examined using residual plots; robust standard errors were employed to ensure valid inference in the presence of heteroscedasticity.

**Fig. 1:**
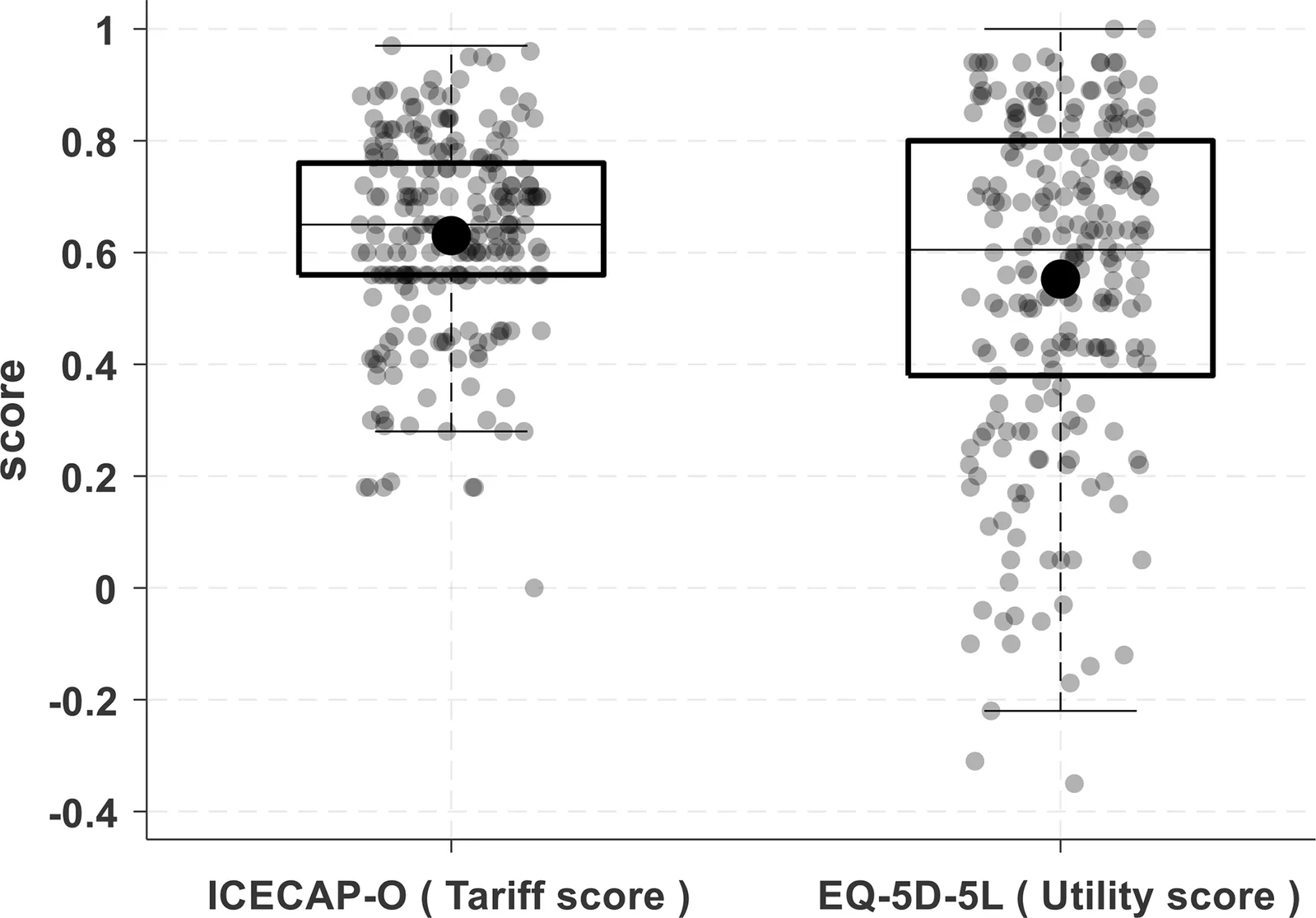
Distribution of ICECAP-O tariff scores and EQ-5D-5L utility scores (*N* = 222). All individual data points are overlaid as semi-transparent black dots to show the full distribution. Black circles represent the mean value for each instrument

Given the non-normal distribution of the data, descriptive statistics are presented as medians (Q1, Q3) throughout the main text and tables; means are shown in Fig. 1 solely to illustrate the distributional shape.

## Results

### Descriptive analysis

Sample characteristics are summarized in Table 1. A total of 222 participants were included in this study. Age ranged from 65 to 93 years [median (Q1, Q3): 72.5 (68.0, 79.0)]. Of these, 116 (52.3%) were female. Most had a primary school education or less (72.1%), were married (62.2%), and 25.2% lived alone. Regarding disease severity, 93.7% were classified as stage II or milder, and 6.3% as stage III. Median disease duration was 30 years (Q1, Q3: 20, 35) and median BMI was 22.04 kg/m² (Q1, Q3: 20.41, 24.03).

**Table 1:** ICECAP-O and EQ-5D-5L scores according to sociodemographic characteristics and clinical conditions in patients with Kashin-Beck disease (*N* = 222)

Variable	Category	*N*(%)	ICECAP-O tariff scores			EQ-5D-5L utility scores		
			Median(Q1,Q3)	*p*-value	Effect size (*r* / η²)	Median(Q1,Q3)	*p*-value	Effect size(*r* / η²)
Sex	Male	106(47.75)	0.6504(0.5541,0.7750)	0.801	-0.017	0.6640(0.4275,0.8068)	0.047	-0.133
	Female	116(52.25)	0.6330(0.5558,0.7556)			0.5235(0.2840,0.7813)		
Education level	Illiteracy	55(24.77)	0.5558(0.4090,0.6953)	<0.001	0.074	0.4140(0.1790,0.5620)	<0.001	0.174
	Primary school	105(47.30)	0.6923(0.5558,0.7861)			0.6190(0.4135,0.7685)		
	Junior high or above	62(27.93)	0.6642(0.6004,0.7846)			0.7665(0.5778,0.8655)		
Marital status	Unmarried/Divorced/Widowed	84(37.84)	0.5558(0.4413,0.7237)	<0.001	-0.277	0.4315(0.2023,0.6465)	<0.001	-0.386
	Married with spouse	138(62.16)	0.6827(0.5968,0.7921)			0.6990(0.5045,0.8480)		
Living alone	No	166(74.77)	0.6827(0.5558,0.7825)	<0.001	-0.273	0.6330(0.4293,0.8280)	0.036	-0.140
	Yes	56(25.23)	0.5558(0.4118,0.6465)			0.5245(0.2270,0.7235)		
Disease severity	Grade I	152(68.47)	0.6764(0.5558,0.7901)	<0.001	0.105	0.6390(0.4310,0.8300)	0.002	0.056
	Grade II	56(25.23)	0.6330(0.4541,0.7237)			0.5885(0.2828,0.7668)		
	Grade III	14(6.30)	0.4252(0.1808,0.5661)			0.2840(-0.0785,0.4758)		

Data are presented as median (first quartile, third quartile) for the ICECAP-O tariff scores and EQ-5D-5L utility scores. The *p*-values refer to the significance of differences between subgroups; the Mann-Whitney *U* test was used for variables with two categories, and the Kruskal-Wallis test for those with three or more categories; Effect sizes: *r* for two-group comparisons (Mann-Whitney *U*), *η²* for multi-group comparisons (Kruskal-Wallis *H*)

The distribution of ICECAP-O tariff scores and EQ-5D-5L utility scores is presented in Fig. 1. Median (Q1, Q3) scores are detailed in Table 1, while means are included in Fig. 1 solely to illustrate the distributional shape. For the ICECAP-O, scores did not differ by sex (*p* = 0.801), whereas for the EQ-5D-5L, scores differed by sex (*p* = 0.047). Both instruments showed significant associations with education level, marital status, living alone status, and disease severity. The scores of both instruments were lower in patients who were illiterate, unmarried/divorced/widowed, living alone, or had more advanced disease (especially stage III).

### Response distributions

The response distributions for the five ICECAP-O dimensions are presented in Table 2. At the “no capability” level, Attachment, Role, and Enjoyment had higher proportions than Control and Security. Conversely, at the “full capability” level, Role and Security showed the highest proportions, while Attachment and Enjoyment were the lowest.

**Table 2:** Response distribution across dimensions of the ICECAP-O (*N* = 222)

Attributes	Level 4		Level 3		Level 2		Level 1	
	*n*	(%)	*n*	(%)	*n*	(%)	*n*	(%)
Attachment	3	1.4	79	35.6	119	53.6	21	9.5
Security	23	10.4	94	42.3	95	42.8	10	4.5
Role	31	14.0	59	26.6	108	48.6	24	10.8
Enjoyment	3	1.4	35	15.8	161	72.5	23	10.4
Control	7	3.2	64	28.8	138	62.2	13	5.9

Each of dimension in ICECAP-O can take one of four levels ranging from full capability to no capability, where level 4 represents full capability and level 1 represents no capability

For the EQ-5D-5L (Table 3), the “no problems” response was most frequent in the Self-care dimension (38.3%, *n* = 85). The response category “extreme problems/unable to” was the least common overall (0.9%, *n* = 2).

**Table 3:** Response distribution across dimensions of the EQ-5D-5L (*N* = 222)

Attributes	Level 1		Level 2		Level 3		Level 4		Level 5	
	*n*	(%)	*n*	(%)	*n*	(%)	*n*	(%)	*n*	(%)
Mobility	59	26.6	64	28.8	57	25.7	31	14.0	11	5.0
Self-care	85	38.3	88	39.6	41	18.5	6	2.7	2	0.9
Usual activities	39	17.6	66	29.7	72	32.4	37	16.7	8	3.6
Pain/ Discomfort	16	7.2	92	41.4	95	42.8	13	5.9	6	2.7
Anxiety/ Depression	53	23.9	62	27.9	71	32.0	30	13.5	6	2.7

Each of dimension in EQ-5D-5L can take one of five levels ranging from no problem to severe problem/ unable to, where level 1 represents no problems and level 5 represents severe problem/ unable to

### Floor and ceiling effects

Floor and ceiling effects were defined as present if more than 15% of respondents reported the lowest or highest possible score, respectively. Based on this criterion, no floor or ceiling effects were observed for any dimension of the ICECAP-O. For the EQ-5D-5L, ceiling effects (≥ 15% reporting “no problems”) were observed in the Mobility (26.6%), Self-care (38.3%), Usual activities (17.6%), and Anxiety /Depression (23.9%) dimensions. No floor effects were observed in any dimension of the EQ-5D-5L.

### Polychoric correlations between ICECAP-O and EQ-5D-5L

As presented in Table 4, the correlation between ICECAP-O tariff scores and EQ-5D-5L utility scores was *ρ* = 0.656 (*p* < 0.001), falling within the moderate-to-strong range (0.4 ~ 0.7) predicted by Hypothesis 1.

Consistent with Hypothesis 2, the strongest negative correlation was between ICECAP-O Control and EQ-5D-5L Usual activities (*ρ* = −0.760, *p* < 0.001), and the weakest (non-significant) correlation was between ICECAP-O Attachment and EQ-5D-5L Self-care (*ρ* = −0.066, *p* = 0.427). Other dimension pairs showed intermediate correlations generally consistent with expectations.

**Table 4:** Polychoric correlation coefficients between ICECAP-O and EQ-5D-5L

ICECAP-O		EQ-5D-5L Dimension					
		Mobility	Self-care	Usual Activities	Pain/Discomfort	Anxiety/Depression	EQ-5D-5L utility
Dimensions	Attachment	-0.191^*^	-0.066	-0.073	-0.214^**^	-0.400^***^	0.230^**^
	Security	-0.478^***^	-0.325^***^	-0.354^***^	-0.417^***^	-0.621^***^	0.532^***^
	Role	-0.656^***^	-0.518^***^	-0.597^***^	-0.454^***^	-0.620^***^	0.682^***^
	Enjoyment	-0.537^***^	-0.414^***^	-0.484^***^	-0.411^***^	-0.619^***^	0.598^***^
	Control	-0.721^***^	-0.639^***^	-0.760^***^	-0.435^***^	-0.490^***^	0.747^***^
Scores	ICECAP-O tariff	-0.604^***^	-0.458^***^	-0.528^***^	-0.451^***^	-0.638^***^	0.656^***^

^*^p<0.05, ^**^p<0.01, ^***^p<0.001. Unmarked coefficients are non-significant

### Known-groups validity

Table 1 presents the results of group comparisons testing the directional predictions of Hypothesis 3. Non-parametric tests indicated that scores on both instruments differed significantly across groups defined by education level, marital status, living alone status, and disease severity (all *p* < 0.05), For sex, the ICECAP-O showed no significant difference (*p* = 0.801), whereas the EQ-5D-5L showed a significant difference (*p* = 0.047). Specifically, median scores were higher among participants with higher education, those who were married, those not living alone, and those with less severe disease — all consistent with the predicted directions. Effect sizes for these comparisons ranged from small to moderate (|*r|* = 0.017 to 0.386 for two-group comparisons; *η*² = 0.056 to 0.174 for multi-group comparisons). The largest effects were observed for education level (*η*² = 0.074 for ICECAP-O, *η*² = 0.174 for EQ-5D-5L) and marital status (|*r*| = 0.277 for ICECAP-O, |*r*| = 0.386 for EQ-5D-5L), indicating that these sociodemographic factors had the strongest practical relevance for discriminating between subgroups. In contrast, the effect size for sex was negligible for ICECAP-O (|*r*| = 0.017) and small for EQ-5D-5L (|*r*| = 0.133), suggesting that despite the statistically significant *p*-value for EQ-5D-5L (*p* = 0.047), the actual difference was limited.

### Regression analysis

Before interpreting the regression results, we assessed multicollinearity using VIF. The VIF values for all predictors ranged between 1.068 and 2.202, with VIF = 1.388 for age and VIF = 1.206 for disease duration, all substantially below the conventional threshold of 10, indicating no concern for multicollinearity. Residual plots for both regression models indicated that the assumptions of linearity and homoscedasticity were met. Regression analysis results are summarized in Table 5. Sociodemographic characteristics and clinical conditions showed distinct associations with the scores of both instruments. Among sociodemographic factors, being female was associated with a significantly lower EQ-5D-5L utility score than being male (*p* = 0.036). Older age was negatively correlated with the scores of both instruments (all *p* < 0.001). Compared to illiterate participants, both primary school and junior high or above education levels were associated with higher ICECAP-O tariff scores (all *p* < 0.05), while only the junior high or above group had a significantly higher EQ-5D-5L utility score (*p* = 0.039). Living alone was negatively associated only with ICECAP-O tariff scores (*β* = −0.066, *p* = 0.011), whereas being married was positively associated only with EQ-5D-5L utility scores (*β* = 0.070, *p* = 0.026). BMI showed a weak negative association only with ICECAP-O tariff scores (*β* = −0.009, *p* = 0.017).

Among clinical variables, longer disease duration was negatively associated with the scores of both instruments (*p* < 0.01). Compared with Grade I severity, Grade II and Grade III were associated with significantly lower ICECAP-O tariff scores (*p* < 0.01), while only Grade III was associated with a significantly lower EQ-5D-5L utility score (*p* < 0.001).

**Table 5:** Ordinary least squares regression results of ICECAP-O and EQ-5D-5L on sociodemographic characteristics and clinical conditions

Variable	Category (Reference)	ICECAP-O			EQ-5D-5L		
		Coef.	S.E.	*p*	Coef.	S.E.	*p*
Sociodemographic Characteristics							
Sex	Female (Male)	0.026	0.022	0.225	-0.058	0.027	0.036
Age (years)		-0.007	0.002	<0.001	-0.028	0.002	<0.001
Body mass index (kg/m²)		-0.009	0.004	0.017	0.001	0.005	0.914
Education	Primary school (Illiteracy)	0.062	0.026	0.016	0.055	0.032	0.094
	Junior high or above (Illiteracy)	0.078	0.032	0.015	0.083	0.040	0.040
Marital status	Married (Unmarried/Divorced/Widowed)	0.003	0.025	0.905	0.070	0.032	0.027
Living alone	Yes (No)	-0.068	0.026	0.010	-0.005	0.033	0.868
Clinical conditions							
Disease duration (years)		-0.003	0.001	<0.001	-0.004	0.001	0.001
Disease severity	Grade II (Grade I)	-0.064	0.023	0.005	-0.051	0.029	0.078
	Grade III (Grade I)	-0.186	0.041	<0.001	-0.191	0.052	<0.001
Model Statistics							
*R* ^*2*^		0.392			0.640		
*N*		222			222		

Coef. coefficient, S.E. standard error

## Discussion

### Item response distribution and floor/ceiling effects

Ceiling effects differed between the two instruments. No ceiling effects were observed for ICECAP-O, suggesting it can sensitively distinguish varying levels of capability in this group. In contrast, the EQ-5D-5L showed ceiling effects in four of its five dimensions (Mobility, Self-care, Usual activities, and Anxiety/Depression). This pattern implies that the EQ-5D-5L may not fully capture limitations in these health domains, possibly due to long-term adaptation or lowered expectations in elderly KBD patients. Conversely, the ICECAP-O, which measures capability well-being rather than health status alone, was free of such ceiling effects, supporting its complementary value in detecting psychosocial limitations that may be missed by health-focused measures. No floor effects were observed for either instrument.

### Polychoric correlations between ICECAP-O and EQ-5D-5L

Table 4 presents polychoric correlations between the two instruments. The correlation between ICECAP-O tariff scores and EQ-5D-5L utility scores was (*ρ* = 0.656, *p* < 0.001), falling within the moderate-to-strong range predicted by Hypothesis 1 (0.4 < *ρ* < 0.7). As specified in Hypothesis 2, the strongest negative correlation was between ICECAP-O Control and EQ-5D-5L Usual activities (*ρ*=-0.760, *p* < 0.001), and the weakest (non-significant) correlation was between ICECAP-O Attachment and EQ-5D-5L Self-care (*ρ* = -0.066, *p* = 0.427).

The total score correlation (*ρ* = 0.656) is higher than those reported in general elderly populations (e.g., ρ = 0.48 in Hungary [30]) and in type 2 diabetes patients (*r* = 0.116 [31]). We interpret this as a context-specific finding rather than evidence of overlap or redundancy. In KBD — a severely disabling osteoarthropathy — physical limitations (mobility, self-care, usual activities) directly constrain an individual’s capability to achieve attachment, security, role, enjoyment, and control. In Sen’s terms [14], health is a foundational capability that enables other capabilities. When foundational capabilities are severely compromised, the correlation between health and broader capability well-being is expected to be higher [32]. This does not imply that the two instruments measure the same construct; rather, it highlights that the degree of overlap is disease-dependent.

The near-zero correlation between Attachment and Self-care confirms that the ICECAP-O captures psychosocial dimensions — specifically, the ability to give and receive love and friendship — that are not reflected in the EQ-5D-5 L, supporting their complementary use [18, 19]. Notably, the stronger health-capability association in KBD compared with type 2 diabetes [31] suggests that disease type may influence the extent to which health status constrains capability well-being. We note that traditional convergent validity requires comparison with a gold-standard instrument; our analysis is not intended as such. Instead, we tested whether the observed correlation fell within the theoretically predicted range (0.4 < *ρ* < 0.7), which is a test of theoretical coherence.

### Known-groups validity and regression analyses

Both instruments showed differences across subgroups (Table 1) that were consistent with the directional predictions of Hypothesis 3, with effect sizes ranging from small to moderate. The larger effects observed for education level (*η*² = 0.074 for ICECAP-O, *η*² = 0.174 for EQ-5D-5L) and marital status (|*r*| = 0.277 for ICECAP-O, |*r*| = 0.386 for EQ-5D-5L) suggest that these sociodemographic factors have stronger practical relevance for distinguishing subgroups of KBD patients. In contrast, the effect sizes for sex were small (|*r*| values ranged from 0.017 to 0.133), illustrating the added value of reporting effect sizes beyond reliance on significance tests alone.

Multiple regression provided further insight into their differential sensitivity. After adjustment for covariates, living alone was independently associated with lower ICECAP-O tariff scores (*β* = −0.066, *p* = 0.011), while being married was associated with higher EQ-5D-5L utility scores (*β* = 0.070, *p* = 0.026). This suggests that social isolation may directly affect the pursuit of life goals (measured by ICECAP-O), whereas spousal support may enhance health-related quality of life (measured by EQ-5D-5L), possibly by providing practical daily assistance.

Moreover, compared with grade I severity, grade II KBD was associated with significantly lower ICECAP-O tariff scores (*β* = −0.064, *p* = 0.006) but did not show a statistically significant effect on EQ-5D-5L utility scores. These findings imply that the capability-based ICECAP-O may be more sensitive than traditional health-focused measures to early declines in psychosocial and functional status in KBD, detecting deterioration in areas such as role, enjoyment, and control before conventional health indicators show marked change.

The known-groups validity analysis also revealed differential sensitivity between the two instruments. The effect size for disease severity was larger for the ICECAP-O (*η*² = 0.105) than for the EQ-5D-5L (*η*² = 0.056), suggesting that the ICECAP-O may be more sensitive in differentiating patients across KBD severity grades. This pattern is consistent with the regression findings, where Grade II severity was significantly associated with lower ICECAP-O scores but not with EQ-5D-5L scores, reinforcing the ICECAP-O’s complementary role in capturing the broader burden of KBD.

Regarding sex, the EQ-5D-5L showed a statistically significant difference (*p* = 0.047), but the effect size was small (|*r*| = 0.133), indicating limited practical relevance. The ICECAP-O showed no significant difference (*p* = 0.801, |*r*| = 0.017). This underscores the importance of interpreting effect sizes alongside p-values, rather than overinterpreting statistically significant but practically small differences.

### Study limitations

This study has several limitations. First, the cross-sectional design precludes causal inference and does not allow assessment of test-retest reliability or the responsiveness of the instruments to changes in health status over time. Therefore, the results of this study should be interpreted with caution when used in economic evaluations, such as cost-effectiveness analyses that require data on changes in health status before and after an intervention. Second, the ICECAP‑O scores were derived using UK-based preference weights; thus, their applicability in the Chinese cultural context requires further validation. Third, because participants were recruited exclusively from a mountainous area of Xi’an, the sample lacks geographic and socioeconomic diversity, which may limit the generalizability of the findings to other KBD-endemic regions (e.g., Tibet, Qinghai). Additionally, all participants volunteered to take part, which may introduce selection bias (e.g., those who agreed to participate might have had greater health awareness), thereby affecting the generalizability of the results.

Future research should employ longitudinal designs to evaluate the responsiveness of the ICECAP‑O and EQ‑5D‑5L in KBD patients, develop Chinese-specific preference weights, and validate the instruments in broader, more representative KBD-endemic regions.

## Conclusions

This study presents the first application of the ICECAP-O instrument in older Chinese patients with KBD. The instrument was free from floor or ceiling effects. While the EQ-5D-5L primarily captured physical health, the ICECAP-O additionally measured psychosocial and capability dimensions. These findings support the complementary use of both instruments to achieve a more comprehensive assessment of patient well-being in this population, thereby informing health economic evaluations.

## Data Availability

The data that support the findings of this study are available from the corresponding author upon reasonable request. The data are not publicly available due to privacy and ethical restrictions.

## References

[1] Wang J, Zhao S, Yang L, et al. Assessing the health loss from Kashin-Beck disease and its relationship with environmental selenium in Qamdo district of Tibet, China. Int J Environ Res Public Health. 2021;18(1):11. 10.3390/ijerph18010011. PMC7792790 33375039

[2] Xu J, Wang J, Zhao H. The Prevalence of Kashin-Beck Disease in China: a Systematic Review and Meta-analysis. Biol Trace Elem Res. 2022;200(12):5229–38. 10.1007/s12011-022-03417-x. 36104539

[3] Hinsenkamp M. Kashin-Beck disease. Int Orthop. 2001;25(3):133–6. 10.1007/s002640000177. PMC3939183 11482524

[4] Xiong G. Diagnostic, clinical and radiological characteristics of Kashin-Beck disease in Shaanxi Province, PR China. Int Orthop. 2001;25(3):147–50. 10.1007/s002640100248. PMC3620652 11482528

[5] Farooq U, Guo X, Chuang LH et al. Measuring health-related quality of life in Kashin–Beck disease using EQ-5D. Qual Life Res 2011;20(3):425–9. doi 10.1007/s11136-010-9820-4. 21184186

[6] Cui S, Que W, Jiao Z, et al. Disease and economic burden of Kashin-Beck disease — China, 2021. China CDC Weekly. 2024;6(2):40–44. 10.46234/ccdcw2024.009. PMC10797301 38250701

[7] Jin Z, Wu X, Sun ZM, et al. Health-related quality of life in patients with Kashin–Beck disease is lower than in those with osteoarthritis: a cross-sectional study. J Orthop Surg Res. 2023;18:236. 10.1186/s13018-023-03803-8. PMC10161486 37143055

[8] National Health Commission. China health statistics yearbook 2022. [M] Beijing: China Union Med Coll Press, 2022: 270 (In Chinese).

[9] Li XX, Fang H, Yang L, et al. Analysis of quality of life and its influencing factors in patients with Kashin-Beck disease. Chin J Endemiology 2020;39(10):709–13. doi 10.3760/cma.j.cn231583-20190423-00113 (In Chinese).

[10] Fei XL, Chen XY, Wang YL et al. Comparative application of WOMAC, SF-36 scales and the criteria for evaluation of therapeutic effect on Kashin-Beck Disease (WS/T 79-2011) in the efficacy evaluation of Kashin-Beck disease patients. Chin J Endemiology 2023;42(8):618–22. 10.3760/cma.j.cn231583-20220310-00071 (In Chinese).

[11] Xia CT, Yu FF, Fang H, et al. Reliability and validity evaluation of the Western Ontario and McMaster Universities Osteoarthritis Index applied to Kashin-Beck disease. Chin J Endemiol. 2015;34(4):278–281. 10.3760/cma.j.issn.2095-4255. 2015.04.011 (In Chinese).

[12] Fang H, Guo X, Farooq U, et al. Development and validation of a quality of life instrument for Kashin-Beck disease: an endemic osteoarthritis in China. Osteoarthr Cartil. 2012;20(7):630–7. 10.1016/j.joca.2012.03.004. 22430051

[13] Liu Y, Wen Y, Yang Z, et al. Prevalence and risk factors of depression in patients with endemic osteoarthritis Kashin–Beck disease. Depress Anxiety. 2025;2025(8722395). 10.1155/da/8722395. PMC12440642 40963707

[14] Sen AK. Capability and well-being. In: Nussbaum MC, Sen A, eds. The Quality of Life. Oxford: Clarendon Press; 1993:30–53.

[15] Al-Janabi H, Flynn TN, Coast J. Development of a self-report measure of capability wellbeing for adults: the ICECAP-A. Qual Life Res 2011;21(1):167–76. 10.1007/s11136-011-9927-2. PMC3254872 21598064

[16] Coast J, Flynn TN, Natarajan L, et al. Valuing the ICECAP capability index for older people. Soc Sci Med 2008;67(5):874–82. 10.1016/j.socscimed.2008.05.015. 18572295

[17] Bibi I, Polman R, Nyman SR. Reliability of the ICECAP-O Quality of Life Scale With Community-Dwelling People With Dementia. Gerontology & Geriatric Medicine. 2022;8:23337214221086802. 10.1177/23337214221086802. PMC8943464 35340365

[18] Davis JC, Liu-Ambrose T, Richardson CG, Bryan S. A comparison of the ICECAP-O with EQ-5D in a falls prevention clinical setting: are they complements or substitutes? Quality of Life Research. 2013;22(5):969–977. 10.1007/s11136-012-0225-4. PMC3672090 22723152

[19] Van Leeuwen KM, Bosmans JE, Jansen APD, et al. Comparing measurement properties of the EQ-5D-3L, ICECAP-O, and ASCOT in Frail Older Adults. Value Health. 2015;18(1):35–43. 10.1016/j.jval.2014.09.006. 25595232

[20] Milte R, Crotty M, Miller MD, et al. Quality of life in older adults following a hip fracture: an empirical comparison of the ICECAP-O and the EQ-5D-3L instruments. Health and Quality of Life Outcomes. 2018;16(1):134. 10.1186/s12955-018-1005-9. PMC6126006 30185193

[21] Ashe MC, Grover S, Bryan S, Cook WL, Donaldson MG, Brasher PMA. Perceived health status and capability after hip fracture: secondary outcomes from a randomized controlled trial. Gerontology. 2024;70(3):235–240. 10.1159/000536131. 38185111

[22] Nakamura CA, Mitchell PM, Peters TJ, et al. A validation study of the EQ-5D-5L and ICEpop capability measure for older people among older individuals with depressive symptoms in Brazil. Value in Health Regional Issues. 2022;30:91–99. 10.1016/j.vhri.2021.11.005. 35325703

[23] Wang J, Wang XY, Li HR, Yang LS, Li YC, Kong C. Spatial distribution and determinants of health loss from Kashin-Beck disease in Bin County, Shaanxi Province, China[J]. BMC Public Health. 2021(21): 387. 10.1186/s12889-021-10407-6. PMC7893884 33607974

[24] Ministry of Health of the People’s Republic of China. Diagnosis of Kashin-Beck Disease (WS/T 207–2010). Beijing: China Standards; 2010.

[25] GREWAL I, LEWIS J, FLYNN T et al. Developing attributes for a generic quality of life measure for older people: preferences or capabilities?[J]. Social Science & Medicine. 2006;62(8): 1891–1901. 10.1016/j.socscimed.2005.08.023. 16168542

[26] Xiong Y, Wu H, Xu J. PCR148 Validation of the Chinese version of the Icecap-O questionnaires among older people with disabilities[J]. Value in Health. 2023;26(6):S339-S339. 10.1016/j.jval.2023.03.1924.

[27] Flynn T N, Chan P, Coast J, et al. Assessing quality of life among British older people using the ICEPOP CAPability (ICECAP-O) measure[J]. Applied Health Economics and Health Policy. 2011;9(3): 167–180. 10.2165/11594150-000000000-00000. 21875162

[28] Herdman M, et al. CDevelopment and preliminary testing of the new five-level version of EQ-5D (EQ-5D-5L)[J]. Quality of Life Research. 2011. 10.1007/s11136-011-9903-x. PMC3220807 21479777

[29] Luo N, Liu G, Li M, et al. Estimating an EQ-5D-5L value set for China[J]. Value in Health. 2017;20(4):662–669. 10.1016/j.jval.2016.11.016. 28408009

[30] Baj P, Farkas M, Dobos A, et al. Capability of well-being: validation of the Hungarian version of the ICECAP-A and ICECAP-O questionnaires and population normative data[J]. Quality of Life Research. 2020;29(6): 1525–1539. 10.1007/s11136-020-02542-1. PMC7561558 32468403

[31] Xiong Y, Wu H, Xu J. Assessing the reliability and validity of the ICECAP-A instrument in Chinese type 2 diabetes patients. Health Qual Life Outcomes 2021;19:28. 10.1186/s12955-020-01632-5. PMC7788876 33407525

[32] Franklin M, Payne K, Elliott RA. Quantifying the relationship between capability and health in older people: Can’t Map, Won’t Map. Medical Decision Making. 2018;38(1):79–94. 10.1177/0272989X17732975. 29056069

